# A Cross-Sectional Study of Professional Nurses’ Knowledge, Attitudes, and Practices Regarding Organ Donation in Critical Care Units of Public and Private Hospitals in the Eastern Cape, South Africa

**DOI:** 10.3390/nursrep13010024

**Published:** 2023-02-13

**Authors:** Bukelwa Green, Daniel Ter Goon, Tobeka Mtise, Olanrewaju Oladimeji

**Affiliations:** 1 Health Professionals Training & Development, Eastern Cape Department of Health, Bisho 5605, South Africa; 2 Department of Nursing, Faculty of Health Sciences, University of Fort Hare, East London 5201, South Africa; 3 Department of Public Health, Faculty of Health Sciences, Walter Sisulu University, Mthatha 5099, South Africa

**Keywords:** organ donation, transplantation, knowledge, attitudes, practice, professional nurses, Eastern Cape

## Abstract

Background: Globally, there is an overwhelming increase in the number of patients waiting for donated organs for transplantation, with a gross shortage of available organs. Lack of clear practice guidelines and the knowledge and attitudes of health care providers were hypothesized as possible reasons. We aimed to determine the attitudes, level of knowledge, and practices of professional nurses working in critical care units in public and private hospitals in Eastern Cape Province regarding organ donation. Method: The study used a quantitative, non-experimental, descriptive design to investigate the current knowledge, attitude, and practice of organ donation in critical care among 108 professional nurses working in public and private critical care units in Eastern cape. Data were collected between 26 February 2017 until 27 June 2017 using anonymous, self-administered, pretested questionnaires. The means of knowledge, and practice scores were estimated among participants, and their associated categorical explanatory variables were ascertained. Results: A total of 108 nurses participated in the study. Of these, 94 (87.0%) were female, 78 (72.2%) were black, 104 (96.3%) were Christians, 79 (73.2%) worked in an ICU, 79 (73.2%) had a diploma qualification, and 67 (62.0%) worked in a tertiary hospital. About 67% of the respondents had good knowledge, 53% had a positive attitude, and 50.4% had poor practice readiness toward organ donation. Working in renal units (*p* < 0.001) and practicing in tertiary hospitals (*p* < 0.001) were significantly associated with a high organ donation knowledge score while being a female nurse (*p* = 0.036), working in renal units (*p* < 0.001), and practicing in tertiary hospitals (*p* < 0.001) were significantly associated with a high organ donation practice score. Conclusion: Differences in organ donation knowledge and practices were noted between the different levels of health care services as the tertiary level outperformed the secondary level institutions. Nurses play a major role in critical and end-of-life care and are closer to patients and relatives. Hence, pre- and in-service education and promotional campaigns among nurses at all levels of care would be a strategic step to scale availability of donated organs and would meet the needs of thousands of individuals who need them to survive.

## 1. Background

Organ transplantation is a therapy or treatment option that saves lives and relieves life-threatening suffering in persons with organ failure. The majority of patients with end-stage organ failure die while still waiting for compatible donors due to the scarcity of donated organs [1]. In the United States, 120,000 people are on the wait list of organ donation while 1000 persons die yearly while waiting for organ donation in Germany [2]. The latest figures from the Organ Donation Foundation shows that there are over 3500 South Africans waiting for organ and tissue transplants [3]. Sadly, due to a critical shortage of donated organs, fewer than 1000 of them will receive organ transplants [3]. A previous study attributed the scarcity of transplantable organs to the ignorance of health care practitioners, and their reluctance to approach families of brain-dead patients for possible donations [4]. The findings could possibly be due to ethical dilemma regarding organ donation. In Eastern Cape Province, there is no organ transplant facility in either public or private institutions, although there are organ transplant coordinators in tertiary hospitals. Anecdotal evidence indicates that critical care units lack the necessary resources to manage organ donation transparently and efficiently, which results in organ transplants being conducted outside the province. A lack of resources should not prevent utilizing knowledge and skills to promote organ donation. Many questions arise regarding organ donation practice in the Eastern Cape, based on uncertainties and lack of knowledge. Nurses are essential members of the team in the donation process, linking the hospital to the organ procurement organization, physicians, and families of potential donors. Their attitudes and levels of knowledge are therefore critical to the success of the process. In addition, the role of critical care nurses in the process of organ donation is not well defined. Before this research was conducted, the attitudes, level of knowledge, and practices among critical care nurses regarding the process of organ donation in public and private hospitals in the Eastern Cape were a matter of speculation. In Turkey, a study showed that understanding and knowledge of organ donation were found to be limited [5]; inadequate knowledge of organ donation was reported among Iranian nurses [6] while health science students lacked knowledge about organ donation and transplantation [7,8]. In the United Kingdom, nurses expressed lack of confidence and preparedness while dealing with brain death and unable to promptly identify potential organ donors among ICU patients with catastrophic brain injury [9]. The absence of guidelines for obtaining informed consent has also been reported as a major barrier to organ donation [10]. In India, the inadequate knowledge and poor attitude of medical students toward organ donation and transplantation prompted its inclusion in the training curriculum [11].

Professional nurses play a key role in identifying potential donors, referring them to organ donation coordinating unit and rendering health care that will ensure that organs are transplantable, by keeping them well perfused and healthy. Knowledge deficiency concerning organ donation among professional nurses could therefore have far-reaching implications for shaping the public’s stand on organ donation, and it is likely to be detrimental. The findings of the KAP study concerning organ donation and transplantation are vital to informing the process of recruiting potential donors. The findings may also inform health policy or management decisions concerning interventions to create awareness among professional nurses on organ donation in the health care system. Therefore, this study was designed to determine the level of knowledge, attitudes, and practices of professional nurses regarding organ donation in private and public hospitals in Eastern Cape Province.

## 2. Methods

### 2.1. Study Design

A quantitative, explanatory, and descriptive study was conducted in specialized units, namely, eight intensive care and three renal care units of public and private hospitals in Eastern Cape Province.

### 2.2. Study Setting

The study was conducted in regional and tertiary public and private hospitals with functional intensive care and renal care units in Eastern Cape Province. The researcher collected data from professional nurses working in the intensive care and renal care units of public and private hospitals in Eastern Cape Province. The hospitals were Frontier Regional Hospital (pilot site), Cecilia Makiwane Regional Hospital, Frere Tertiary Hospital, Livingstone Tertiary hospital, Nelson Mandela Tertiary/Academic Hospital, Life St Dominic’s Hospital, and Life Queenstown Hospital. The targeted population in this study was professional nurses working in intensive care and renal care units, trained in critical care or nephrology, as well as those who were not trained but worked in these specialized areas.

### 2.3. Study Size

A total of 108 professional nurses were included in this study.

### 2.4. Sampling Technique

The study utilized purposive sampling, based on an assessment of whether participants were knowledgeable about the question at hand [12]. The total population in this study constituted 108 professional nurses working in public and private critical care units. All professional nurses in critical and renal care were qualified to participate in the study due to the fact that there are very few such critical and renal care units in the Eastern Cape Province. Not all the hospitals have such units.

### 2.5. Inclusion and Exclusion Criteria

Participants were included if they were trained and experienced professional nurses, working in critical care and/or renal care units, and believed to have sufficient knowledge of the phenomenon under study. Participants were excluded from the study if they were not professional nurses, irrespective of experience.

### 2.6. Bias

An open-ended question was used for this study to avoid the social desirability bias on the participants’ knowledge, attitude, and practices regarding organ donation.

### 2.7. Data Collection

Data were collected using a self-administered questionnaire. Data were collected between the 26 February 2017 and 27 June 2017. The questionnaire was divided into four sections: Section A focused on a demographic profile of the participants, Section B solicited information concerning the availability of standardized procedures and practices regarding organ donation in the unit, Section C comprised questions on the knowledge of professional nurses on organ donation, and Section D addressed professional nurse’s attitudes in relation to organ donation. Sections A, B, C, and D contained 7, 13, 15 and 10 questions, respectively. The attitude questions were framed in Likert scale format and the respondents had the opportunity to choose among strongly disagree, disagree, neutral, agree, and strongly agree options. A pretest of the questionnaire was conducted on ten professional nurses in order to identify gaps and ambiguous wording in the questionnaire. The results of the pretest indicated no ambiguity or difficulty among participants when filling in the questionnaire. Participants were given the opportunity to read and sign an informed consent form before participating in the study. The aim and the nature of the study were explained prior to commencement. Given that specialized units are one of the busiest units in the hospital and require close and continuous monitoring of the patients, and to avoid disturbing service delivery, data were collected during tea times and lunch breaks. Anonymity was maintained because the questionnaires were only coded without requiring the names of candidates. The researcher observed all ethical considerations in the study by complying with ethical principles of nursing research, and the ethical requirements of the University of Fort Hare, the Eastern Cape Department of Health, and the targeted public and private hospitals. Permission was sought in writing from hospital management to access the facilities and it was granted. The participants were given information about the nature of the study and understood that they had the option to participate or not.

### 2.8. Variables

The outcome/dependent variables were knowledge and practice scores regarding organ donation. Independent variables include participants’ practice unit (ICU or renal), race (black or others), qualification (diploma or degree), gender, hospital category (tertiary, regional, and private), hospital specialty (no specialty, critical care, and nephrology). Study variables were presented using descriptive statistics such as frequency and percentage. Knowledge, attitude, and practice were categorized and presented as good/poor, positive or negative, and good or poor practice, respectively. The knowledge and practice scores were estimated among participants, and their associations with categorical explanatory variables were ascertained through the student’s t-test or one-way ANOVA as applicable.

### 2.9. Data Sources/Measures

A 45-item self-administered questionnaire comprising four sections was developed based on previous studies [13,14]. The first section, Q1–7 of the questionnaire, comprises the demographic details of the study participants, which include gender, age, religion, race, qualification, years of practice as a professional nurse, and years of practicing as a specialist. The second, third, and fourth sections assessed the practice of organ donation procedure (Q8–22), nurses’ knowledge regarding the donation process (Q23–35), and nurses’ attitude toward organ donation (Q36–45), respectively. The responses were recorded on a dichotomous scale (Yes/No). Each “Yes” response was scored “1” and each “No” response “0.” The attitude scale was designed in Likert format (Q36–Q45). The total scores obtained were summed up. The higher scores indicated good knowledge, positive attitude, and good practice while the lower score indicated poor knowledge, negative attitude, and poor practice habits regarding organ donation. The proportion of respondents whose performance fell within a scoring category was calculated and presented as a percentage.

### 2.10. Data Analysis

Data were captured in Excel and exported for analysis using the Statistical Package for Social Sciences (SPSS) version 22. Descriptive and inferential statistical analyses were performed. Frequencies and percentages were used for describing the categorical socio-demographic variables. Independent sample and student’s t-test were used for comparing practice and knowledge across race, gender, unit, and qualification. For comparing unit practice and knowledge scores across the different hospital categories and specialties, the one-way analysis of variance (ANOVA) was used. This was followed by Tukey’s post hoc analysis. A *p*-value of 0.05 was considered statistically significant.

## 3. Results

The total targeted number of professional nurses was 187, of which 108 responded to the questionnaires. In total, 64 questionnaires were returned incomplete/wasted, giving a response rate was 108/187 (58%). A total of 108 nurses participated in the study. Of these, 94 (87.0%) were female, 78 (72.2%) were black, 104 (96.3%) were Christians, 79 (73.2%) worked in ICU, 79 (73.2%) had a diploma qualification, and 67 (62.0%) worked in a tertiary hospital. The distribution of participants by biographical characteristics is presented in Table 1.

However, only 68 (62.7%) respondents had adequate knowledge about organ donation; 57 (53%) had a positive attitude (strongly agree and agree) and 54 (49.7%) respondents had a good practice/readiness score toward organ donation (as shown in Table 2).

Males scored lower than females on both unit practice and self-evaluated knowledge. However, only the difference for the unit practice scores was found to be statistically significant (t = −2.2; *p* = 0.036), while no significant differences were detected for self-evaluated knowledge (t = −0.5; 0.602). Comparisons with respect to the unit showed that ICU nurses scored lower than renal nurses on both unit practice and self-evaluated knowledge. The tests for statistical significance showed that the differences on both variables were statistically significant, with t = −5.7; *p* < 0.001 and t = −3.3; *p* = 0.001 for unit practice and self-evaluated knowledge, respectively (as shown in Table 3).

Tertiary hospitals had the highest mean practice (68.4%) and knowledge (67.9%) scores. The hospital category was found to significantly affect both the unit practice (F = 53.4; *p* < 0.001) and knowledge (F = 9.4; *p* < 0.001). The tests for statistical significance show that the practice scores are not significantly different across the three specialties (F = 2.5; *p* = 0.088), while the knowledge score differences are borderline significant (F = 3.1; *p* = 0.050) (as shown in Table 4).

## 4. Discussion

The study aimed to investigate the knowledge, attitude, and practice of professional nurses and associated factors toward organ donation in Eastern Cape, South Africa. The study observed that only 62.7%, 53%, and 49.7% of professional nurses had good knowledge, positive attitude and good practices toward organ donation, respectively. These findings point to a knowledge deficit among nurse professionals and could be the outcome of limited or non-existing organ donation modules in nursing training curricula and in workplace in-service education programs, most especially for those practicing in secondary and regional hospitals. Lack of knowledge regarding practice of donation would indirectly compromise the quality of care to be rendered to the patient and will eventually break the connection between nurses and the patient.

Additionally, working in renal units and tertiary hospitals was significantly associated with higher organ donation knowledge score while being a female nurse, and working in renal units and practicing in tertiary hospitals were significantly associated with higher organ donation practice score. Similar findings were reported in Hyderabad India, where female subjects had significantly higher knowledge, practice, and attitude scores [15,16], as well as in Saudi Arabia, where female participants scored significantly higher in the domain of knowledge, attitude and practice toward organ donation [17]. These could be a result of women being emotional, empathetic, caring, sacrificial, and with higher professional values (compared to men) [18], an attribute that dictates societal direction toward practices such as organ donation. However, in contrast, data on the association of gender with organ donation practice in Saudi Arabia noted that male adult respondents had better knowledge than females [19]. Since both males and female nurses have equal chance of displaying knowledge, a possible explanation could be higher number of women in this study compared to men (87 versus 13%).

Tertiary hospitals were found to be more exposed to the practice (68.4%), with more knowledge (67.9%) regarding organ donation than those in regional hospitals regarding practice (13.8%) and knowledge (43.3%). Tertiary hospitals offer specialized and higher levels of care, often dealing with multi-organ failure and brain-dead patients, whereas regional hospitals often refer to tertiary hospitals. Contrary to the findings of this study, ICU nurses at local (district) hospitals in Sweden reported a more positive attitude toward organ donor advocacy overall compared with nurses at larger regional or university hospitals [20]. The investigators explained this by stating that at tertiary hospitals, staff turnover is higher, and nurses are usually younger than at district hospitals, and therefore less experienced and knowledgeable than those in the smaller district hospitals.

Context specific differences may also explain some of the findings. In contrast to South Africa, for instance, identifying organ donor in the United Kingdom was traditionally restricted to the ICU until this was expanded to the emergency department, which now works with specialist nurses for organ donation (SN:OD) following the UK Organ Donation Taskforce report in 2008 [21]. In addition, renal care units and nurses with nephrology qualifications were more exposed to the practice and had more knowledge than ICU nurses did. Renal failure is one of the leading end-stage organ failures in South Africa and in the Eastern Cape Province, and so kidney donations and transplantations are fairly common since the procedure utilizes live donors. The formal scope of practice for nurses regarding organ donation needs to be clearer, as the lack of clarity in interpreting the roles of nurses in organ donation process could contribute to poor practices and procedures at different levels of care and units.

The study has several strengths and limitations. First, an open-ended question was used for this study to avoid the social desirability bias on the participants’ knowledge, attitude, and practices regarding organ donation. The study population was limited to professional nurses and excluded other levels of nurses and other clinicians responsible for organ donation simply because specialized nursing care is usually offered by professional nurses and not enrolled nurses. The study targeted ICU and renal care units only and excluded emergency units, which are viewed as the entry point for all patients. In addition, self-reporting can be biased and prone to overstatement. Moreover, because this was a cross-sectional study, causal associations could not be ascertained.

The roles of various healthcare professionals are vital in the transplant program since they are on the frontline across different healthcare institutions, which includes hospitals, medical centers, and medical colleges in direct contact with patients. The South African Nursing Council should revise the scope of the practice and key performance areas for professional nurses working in specialized units. All nursing education institutions must revise their pre-registration curricula. Organ donation policies, protocols, and procedure manuals should be developed and made easily accessible to all health professionals. An awareness campaign should ensure that the Organ Donation Foundation’s contact information is shared with all hospitals. The study only included professional nurses who worked in intensive and renal care units. Further research could consider all health professionals involved in organ donation and transplantation, as well as nurses working in emergency departments. Finally, more research should be conducted to investigate the general public’s attitudes toward organ donation.

## 5. Conclusions

The study revealed a clear knowledge deficit with subsequent inadequate practice, but this deficit had no effect on positive attitudes toward the idea. There was a difference between levels of health care services for all three variables, with nurses in tertiary hospitals scoring higher for knowledge, attitude, and practice than nurses in regional hospitals. It is critical for ICU nurses to be actively involved in identifying potential organ donors, as well as to be directly involved in the organ donation process and to be continuously retrained. The issue of reviewing and incorporating organ donation modules into health science curricula came up strongly, which is similar to what is reported in other studies. 

## Figures and Tables

**Table 1:** Demographic characteristics of the participants.

Variables	*n* (%)
Gender	
Male	14 (13.0)
Female	94 (87.0)
Hospital type	
Private	28 (25.9)
Regional	13 (12.0)
Tertiary	67 (62.0)
Hospital	
Cecilia Makiwane	13 (12.0)
Frere	20 (18.5)
Livingstone	29 (26.9)
Nelson Mandela Academic	18 (16.7)
Private Hospital 1	5 (4.6)
Private Hospital 2	15 (13.9)
Private Hospital 3	8 (7.4)
Race	
Black	78 (72.2)
Colored	21 (19.4)
Indian	5 (4.6)
White	4 (3.7)
Religion	
Christianity	104 (96.3)
Other	4 (3.7)
Qualification	
Diploma	79 (73.2)
Degree	29 (26.8)
Unit	
ICU	79 (73.2)
Renal	29 (26.8)
Specialty	
None	46 (42.6)
Critical care	45 (41.7)
Nephrology	15 (13.9)
Both	2 (1.8)

**Table 2:** Percentage of respondents with knowledge, attitude and practice score.

Knowledge	%
Good knowledge	62.7
Poor knowledge	37.3
Attitude	
Strongly disagree	15.6
Disagree	17.0
Neutral	14.4
Agree	29.3
Strongly agree	23.7
Practice	
Good Practice	49.7
Poor Practice	50.4

**Table 3:** Comparisons of organ donation practice and knowledge by unit, race, gender, and qualification.

Variable		N	Mean	Mean Diff	Std. Error	Lower Limit	Upper Limit	t	*p*-Value
Practice	Black	78	44.9	−6.5	4.413	−23.22	10.3	−0.76	0.446
	Other	30	51.3						
Knowledge	Black	78	59.9	2.9	3.277	−9.52	15.22	0.46	0.648
	Other	30	57.1						
Practice	Diploma	79	44.7	−7.4	8.536	−24.31	9.54	−0.87	0.389
	Degree	29	52.1						
Knowledge	Diploma	79	58.4	−2.8	6.305	−15.32	9.68	−0.45	0.656
	Degree	29	61.2						
Practice	Male	14	30	−19.1	8.558	−36.9	1.4	−2.2	0.036
	Female	94	49.1						
Knowledge	Male	14	55.4	−4.4	8.316	−20.84	12.14	−0.5	0.602
	Female	94	59.7						
Practice	ICU	79	35.2	−42.7	7.493	−57.6	27.89	−5.7	0.000
	Renal	29	77.9						
Knowledge	ICU	79	53.8	−19.9	6.008	−31.82	−8.00	−3.3	0.001
	Renal	29	73.7						

**Table 4:** Comparisons of practice and knowledge by hospital specialty post hoc test.

Variable	N	Mean	Std. Error	95% CI	F	*p*-Value	Homogeneous Groups
Practice by Hospital Category							
Tertiary	67	68.4	4.171	60.03–76.69	53.4	<0.001	A
Regional	13	13.8	2.665	8.04–19.65			B
Private	28	10	2.242	5.40–14.60			B
Knowledge by Hospital Category							
Tertiary	67	67.9	3.549	60.82–75.00	9.4	<0.001	A
Regional	13	43.3	4.389	33.71–52.83			B
Private	28	45.5	4.777	35.73–55.34			B
Practice by Hospital specialty							
No specialty	46	42.8	5.87	31.00–54.65	2.5	0.088	A
Critical Care	45	43.3	5.703	31.84–54.83			A
Nephrology	17	65.9	8.955	46.90–84.87			A
Knowledge by Hospital specialty							
No specialty	46	53.5	4.608	44.25–62.81	3.1	0.05	A
Critical Care	45	59.4	3.814	51.76–67.13			AB
Nephrology	17	73.5	6.597	59.54–87.51			B

## Data Availability

The data presented in this study are available on request from the corresponding author. The data are not publicly available due to privacy restrictions.

## Abbreviations

KAP	Knowledge, attitude, and practice
ICU	Intensive Care Unit
SPSS	Statistical Package for Social Sciences
ANOVA	Analysis of Variance
UK	United Kingdom
